# Modeling of Advanced Silicon Nanomaterial Synthesis Approach: From Reactive Thermal Plasma Jet to Nanosized Particles

**DOI:** 10.3390/nano12101763

**Published:** 2022-05-22

**Authors:** Samira Elaissi, Amira Ben Gouider Trabelsi, Fatemah H. Alkallas, Tahani A. Alrebdi, Kamel Charrada

**Affiliations:** 1 Department of Physics, College of Science, Princess Nourah Bint Abdulrahman University, P.O. Box 84428, Riyadh 11671, Saudi Arabia; 2 Research Unit of Ionized Backgrounds and Reagents Studies (UEMIR), Preparatory Institute for Engineering Studies of Monastir (IPEIM), University of Monastir, Kairouan Street, Monastir 5019, Tunisia

**Keywords:** modelling, thermal plasma, fluid dynamic, turbulence, silicon, nanopowder, process control

## Abstract

A three-dimensional numerical modelling of a time-dependent, turbulent thermal plasma jet was developed to synthetize silicon nanopowder. Computational fluid dynamics and particle models were employed via COMSOL Multiphysics^®^ *v.* 5.4 (COMSOL AB, Stockholm, Sweden) to simulate fluid and particle motion in the plasma jet, as well as the heat dependency. Plasma flow and particle interactions were exemplified in terms of momentum, energy, and turbulence flow. The transport of nanoparticles through convection, diffusion, and thermophoresis were also considered. The trajectories and heat transfer of both plasma jet fields, and particles are represented. The swirling flow controls the plasma jet and highly affects the dispersion of the nanoparticles. We demonstrate a decrease in both particles’ velocity and temperature distribution at a higher carrier gas injection velocity. The increase in the particle size and number affects the momentum transfer, turbulence modulation, and energy of particles, and also reduces plasma jet parameters. On the other hand, the upstream flame significantly impacts the particle’s behavior under velocity and heat transfer variation. Our findings open the door for examining thermal plasma impact in nanoparticle synthesis, where it plays a major role in optimizing the growth parameters, ensuring high quality with a low-cost technique.

## 1. Introduction

Nanomaterial particles attracted immense interest in recent decades, owing to their unique characteristics. In particular, the optical, electronic, and catalytic properties change with the matter size, and differ significantly in nanometric scale to those observed in micrometer-sized particles and bulk materials [1,2,3]. Nanosized particles are widely used in various domains such as industry, biomedical, and environmental purification processes [4,5,6,7]. 

Amorphous silicon nanostructures attracted considerable interest for many years, and were extensively used in several domains, such as optoelectronics, photovoltaic, and energy storage [8,9,10]. Indeed, silicon remains one of the most promising candidates known so far in lithium-ion batteries anodes Lithium-ion batteries (LIBs). This is due to its remarkable capacity for storing energy [11].

Various techniques were developed to grow silicon nanomaterials, including chemical vapor deposition, non-thermal or thermal plasma-assisted chemical vapor deposition, and laser ablation [12,13,14]. However, chemical routes still face challenges in obtaining high-quality nanopowders in mass quantities, due to the synthesis initiation starting from the liquid phase, or combustion. On the other hand, silicon nanoparticles synthesis via the non-thermal plasma method is hampered, due to its limited productivity and hazardous precursor (SiH4) [15]. 

Thermal plasma offers a promising method for developing high-quality nanopowder with narrow size distributions [16,17]. Its huge enthalpy and easily managed power lead to fast chemical reactions and rapid heat transfer. Indeed, nanomaterial deriving from thermal plasma growth highly depends on the target materials, as well as the plasma torches used [18]. Transferred (DC) plasma torches were widely utilized to produce nanomaterial powders of 50 nm size, at high yields, and with greatly efficient advanced steps [19,20]. 

Recently, thermal plasma achieved high-throughput fabrication of silicon nanoparticles used for lithium-ion battery electrodes. Indeed, Kambara et al. [21] produce nanocomposite silicon with an output of 6 g/min, based on the use of plasma spraying and silicon powder as a raw material. A plasma jet assisted by direct current is used by Ohta et al. [22] to produce silicon nanoparticles, at a rate of 17 g/min. Zhang et al. [23] and Tanaka et al. [24] synthesize amorphous silicon nanoparticles with inductively coupled thermal plasma (ICTP), using a pulse modulation technique. Despite the widespread scientific and industrial interest in nanoparticle growth using thermal plasma, the mechanism of their formation remains mysterious. A complex process involving both heat and mass transfer occurs during nanoparticle growth via a thermal plasma route, and it involves nucleation, condensation, coagulation, diffusion, convection, and thermophoresis. Indeed, the thermal plasma process is based on several parameters that describe the interaction of the thermo-fluid field and the concentration field of the particles [25]. 

In this regard, the transport of particles requires high control, as well as plasma gas composition, to ensure nanomaterials synthesis with predetermined stoichiometry [26]. However, experimental works remain unable to give a spatial and temporal description of the growth process, and only final product characteristics are evaluated. Furthermore, many experiments are needed to specify the optimum performance of any process in terms of controlling particle size and composition, which demands higher time and cost expenditures. Consequently, thermal reactive plasmas are limited specifically to the growth of nanopowders. High costs, low production volume, and weak control of the structural properties represent the main hindrances in industrial applications. Modelling and computational approaches may solve such difficulties, by presenting an appropriate understanding of nanoparticle synthesis, and defining a lower cost process. Previously, Xiong et al. establish a three-dimensional model to estimate nanoparticles suspensions behavior in spray coatings [27]. They succeed in determining several parameters effects’, such as the droplet size, injection angle, and nanoparticles velocity, on the growth process. On other hand, Shigeta and Watanabe theoretically examine the size distribution and the growth process dependency on the saturation pressure, by simulating metal silicide nanoparticles using a thermal plasma [28]. Later, Shigeta addresses the turbulence effect on thermal plasma flow as it is a major contributor in the generation of nanoparticles. Indeed, turbulence demonstrates a significant mixing effect, affecting both the process qualities and the momentum energy.

Herein, a three-dimensional numerical simulation of nanopowder formation using thermal plasma jet was provided. The velocity of the turbulent flow and the field temperature, as well as the swirl velocity effect on plasma characteristic fields, are examined. Furthermore, several process conditions, such as carrier gas velocity, size, and number of particles, and initial plasma jet properties were optimized. The effect of the variation in swirl flow intensity on the particle’s dispersion was investigated. The studied parameters reveal a high dependency on the agglomeration degree of the synthesized nanoparticles.

## 2. Nanoparticles Synthesis

The plasma reactor system employed for nanopowder growth contains a downward, non-transferred thermal plasma torch, as we reported in our previous work [29]. The cathode is made of thoriated tungsten, while the anode is made from copper. The plasma-forming gas in DC torches is flown with the arc column and heated by the joule effect and radiation heating. 

**Figure 1:**
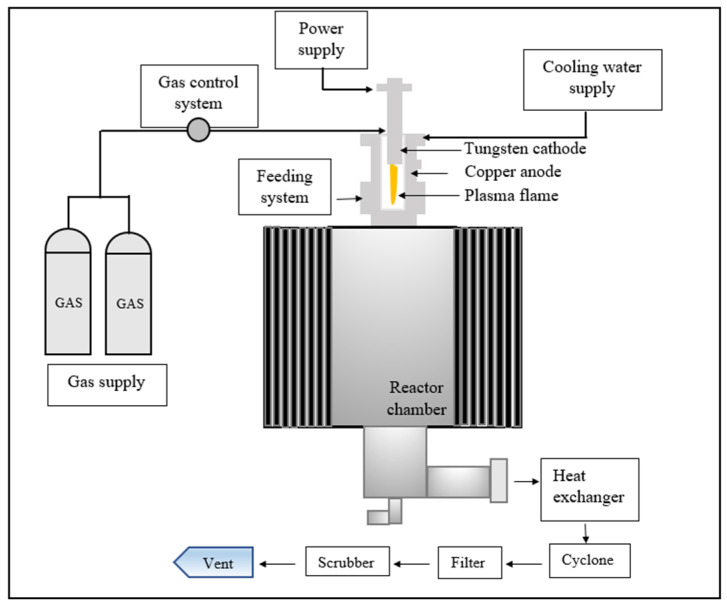
Non-transferred plasma reactor diagram.

Figure 1 illustrates the experimental apparatus of the non-transferred plasma reactor used for nanoparticle formation.

Several inert and reductive gases are used in DC torches, such as Ar, N_2_, H_2_, He, or even a gas mixture. Oxidizing gases are not recommended for such a torch type, where an oxide layer may appear and covert the anode. This reduces the thermal and electric conductivity of the anode, and destruct the electrode [30]. However, argon gas is chemically inert and easily ionized, which makes it a better choice for high-purity growth. This is proven during the fabrication of various sensitive semi-conductor materials [30]. In DC torches, the metallic electrodes are eroded by the arc root attachments. The partially melted or evaporated electrodes occasionally affect the product purity, and the shorter life of the electrode increases the operating cost. However, this could be controlled by adjusting the arc rotation stability [31]. The operating parameters used for silicon nanoparticle synthesis are summarized in Table 1.

**Figure 2:**
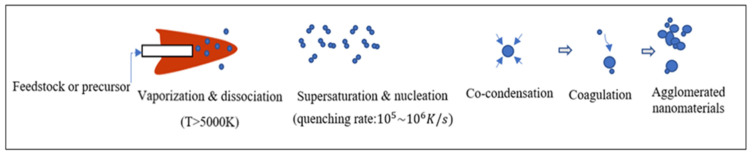
Principle of nanoparticles generation by thermal plasma.

The expanded argon plasma flame or jet is emitted from the plasma torch in the reactor chamber confined by the cooled cylindrical surface. Crystallized silicon particles, around 20 µm in diameter, are added to the high temperature plasma flame through argon carrier gas where they are immediately vaporized. The vapor is then transferred into the rim or tail of the plasma flame and quenched, where the temperature decreases considerably, due to the thermal expansion induced by the high-velocity flow. The silicon vapor becomes supersaturated by the rapid quenching action, and nucleates to form silicon nuclei. Simultaneously, numerous nuclei grow by heterogeneous condensation and coagulation. This coagulation process is vital for the collective development of particles. Consequently, nano-sized particles are generated [32]. The nano-growth of Si particles using thermal plasma is summarized in Figure 2.

## 3. Modeling Approach

**Figure 3:**
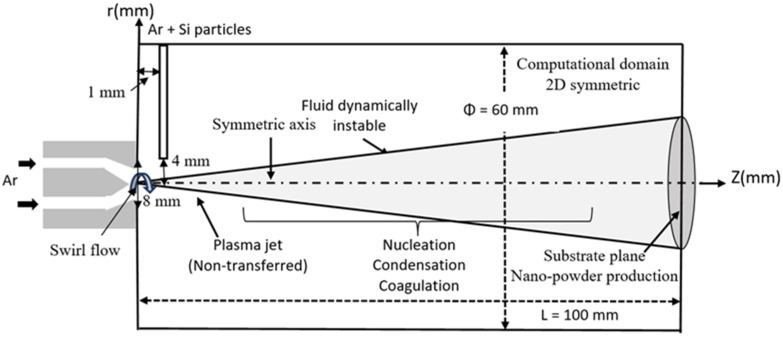
Schematic diagram of nanopowder production with a non-transferred thermal plasma jet.

The thermal argon plasma jet injected by the torch into an argon environment reactor chamber at atmospheric pressure is represented in Figure 3. Particles and carrier gas are delivered into the radical direction, 4 mm away from the center line and 1 mm under the nozzle exit. Here, the thermal plasma jet is established with a non-transferred DC torch [33].

A 3D approach is required in this study, because when particles are injected transversely into the plasma jet, they are assumed to swirl and/or disperse in turbulent ways. Consequently, they take on 3D trajectories that need to be analyzed in 3D geometry, in order to improve accuracy of the numerical model.

### 3.1. Plasma Jet Model

The 3D time-dependent model used in our study could be described by admitting the below assumptions [34]: (a) plasma is optically thin and described in local thermodynamic equilibrium; (b) plasma flow is turbulent because multiscale eddies have a significant impact on the movement of particles; (c) the viscous dissipation could be neglected due to the small pertinent Mach number (less than 0.3); (d) the argon plasma jet is released in argon ambient gas; (e) in plasma flow, transport and thermodynamic characteristics vary with temperature and pressure; and (f) due to a low particle loading rate, the particle injection’s effect is neglected.

Therefore, the following equations are used to simulate thermal plasma jet flow [35]:

Equation of mass conservation:

(1) $$\frac{\partial \rho }{\partial t}+\nabla \cdot (\rho u)=0$$

Equation of momentum:

(2) $$\frac{\partial \left(\rho u\right)}{\partial t}+\nabla \cdot \left(\rho uu\right)=-\nabla P+\nabla \cdot \left\{\Gamma_{\mu }\left[2S-\frac{2}{3}\left(\nabla \cdot u\right)I\right]\right\}$$

Equation of energy:

(3) $$\frac{\partial \left(\rho h\right)}{\partial t}+\nabla \cdot \left(\rho uh\right)=\nabla \cdot \left(\Gamma_{\kappa }\nabla h\right)+Q_{p}-Q_{\mathrm{rad}}$$

where **u**, ρ, h, and P represent the velocity vector, the fluid density, the enthalpy, and the pressure, respectively. $\Gamma_{\mu }$ and $\Gamma_{\kappa }$ denote the effective viscosity coefficient and the effective thermal conductivity, respectively. I is the unit matrix, and $Q_{p}$ and $Q_{\mathrm{rad}}$ are the heat generated from condensation and radiation loss, respectively. S represents the strain tensor velocity, given by:

(4) $$S=\frac{1}{2}\left[\left(\nabla u\right)+{\left(\nabla u\right)}^{\mathrm{tr}}\right]$$

where the (^tr^) indicates the transposition.

The (K−ε) turbulent model is assigned to simulate plasma jet flow, where the turbulent kinetic energy K and the dissipation rate ε are given by [36,37,38]:

Turbulent kinetic energy K:

(5) $$\frac{\partial \left(\rho K\right)}{\partial t}+\nabla \cdot \left(\rho uK\right)=\nabla \cdot \left(\Gamma_{K}\nabla K\right)+G-\rho \varepsilon$$

Turbulent dissipation rate ε:

(6) $$\frac{\partial \left(\rho \varepsilon \right)}{\partial t}+\nabla \cdot \left(\rho u\varepsilon \right)=\nabla \cdot \left(\Gamma_{\varepsilon }\nabla \varepsilon \right)+\frac{\varepsilon }{K}\left(C_{1}G-C_{2}\rho \varepsilon \right)$$

The production term G is defined as:

(7) $$G=\mu_{t}\left[\nabla u:\left(2S\right)-\frac{2}{3}{\left(\nabla u\right)}^{2}\right]-\frac{2}{3}\rho K\nabla \cdot u$$

The coefficients $\Gamma_{\mu }$, $\Gamma_{\kappa }$, $\Gamma_{K}$, and $\Gamma_{\varepsilon }$ are expressed as:

(8) $$\Gamma_{\mu }=\mu +\mu_{t}$$

(9) $$\Gamma_{\kappa }=\kappa +\frac{\mu_{t}C_{p}}{{\mathrm{Pr}}_{t}}$$

(10) $$\Gamma_{K}=\mu +\frac{\mu_{t}}{\sigma_{K}}$$

(11) $$\Gamma_{\varepsilon }=\mu +\frac{\mu_{t}}{\sigma_{\varepsilon }}$$

μ, κ, and $C_{p}$ correspond to the viscosity coefficient, the thermal conductivity, and the specific heat at constant pressure, respectively. $\mu_{t}$ is the turbulent kinematic viscosity as shown below:

(12) $$\mu_{t}={\rho C}_{\mu }\frac{K^{2}}{\varepsilon }$$

The considered values of turbulent constants are: $C_{1}$ = 1.44, $C_{\mu }$ = 0.09, $C_{2}$ = 1.92, ${\mathrm{Pr}}_{t}$ = 0.9, σ_ε_ = 1.3, and σ_k_ = 1 [39]. The thermodynamic properties and transport coefficient of argon plasma are reported from [40]. 

The boundary conditions employed in this model is summarized as follows:

At the jet inlet, (i.e., at the nozzle exit of the non-transferred plasma torch), we admitted the assigned polynomials expression used by M. Shigeta [41], based on the experimental measurement taken from [42], and verified using our numerical model of non-transferred plasma jet studied in our previous work at the same conditions [43].

(13) $$u_{z\;(\mathrm{in})}=150+33\left(\frac{r}{r_{\mathrm{wall}}}\right)-\;614{\left(\frac{r}{r_{\mathrm{wall}}}\right)}^{2}+928{\left(\frac{r}{r_{\mathrm{wall}}}\right)}^{3}-\;480{\left(\frac{r}{r_{\mathrm{wall}}}\right)}^{4}$$

(14) $$T_{\left(\mathrm{in}\right)}=10,400\;-10,500\left(\frac{r}{r_{\mathrm{wall}}}\right)+33,533{\left(\frac{r}{r_{\mathrm{wall}}}\right)}^{2}-\;64,000{\left(\frac{r}{r_{\mathrm{wall}}}\right)}^{3}+33,067{\left(\frac{r}{r_{\mathrm{wall}}}\right)}^{4}$$

**Figure 4:**
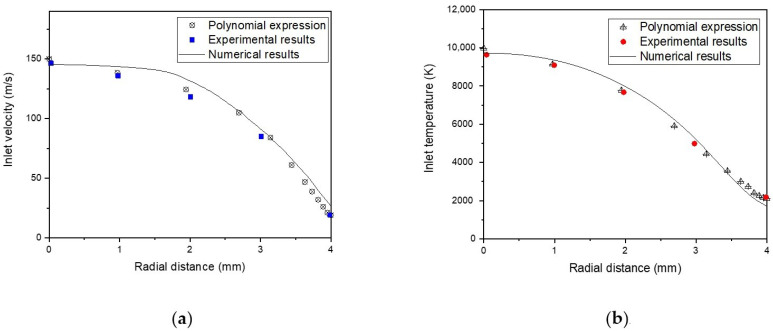
Distribution of velocity (**a**) and temperature (**b**) profiles at the plasma jet inlet as a function of radial distance, using a numerical model, experimental results, and polynomial expression.

A comparison between the numerical calculation, the polynomial expression, and experimental results of axial velocity $u_{\mathrm{in}}\;$and temperature $T_{\mathrm{in}}\;$is illustrated in Figure 4.

The radial velocity at the nozzle exit is not considered, $u_{r}=0$, and the turbulent kinetic energy and dissipation rate are assumed as $K=0.005u_{z}^{2}$, $\varepsilon =0.1K^{2}$, respectively.

In addition, the flow being swirling at the jet inlet and the azimuthal velocity are defined as [44]:

(15) $$u_{\theta }=\{\begin{array}{ll} u_{\theta m}\left(\frac{r}{R_{s}}\right), & z=0,\;r\leq R_{s}\\ u_{\theta m}\frac{R_{s}}{R\;-{\;R}_{s}}\left(\frac{R}{r}-1\right), & z=0,\;r>R_{s} \end{array}$$

where $R_{s}=\left(2/3\right)R$, and $u_{\theta m}=100\;m/s$. A swirling velocity flow, taking a combined form of a free and solid vortex, is assumed at the jet inlet. The pertinent swirl number at the jet inlet is rather small, due to the comparatively high axial momentum flux of the plasma jet, and is defined as:

(16) $$S^{\prime }=\int_{0}^{R}{\rho u}_{z}u_{\theta }r^{2}\mathrm{dr}/\left[R\times \int_{0}^{R}{\rho u}_{z}^{2}\mathrm{rdr}\right]$$

The non-slip condition ($u_{z}$ = $u_{r}$ = 0) on the velocity fields is applied for the walls of the reactor chamber with fixed temperatures (T = 300 K).

$u_{z}$ and $u_{r}$ are the axial and radial velocity vectors, respectively.

Finally, Newman conditions are assumed, applicable to all variables at the outlet boundary of the computation domain, satisfying the conservation of the mass flow [45].

### 3.2. Nanopowder Transport Model

We considered the following assumptions to investigate particle transport and growth [46]: (a) we neglected the electric charge effects while the vaporized material is considered as an ideal gas; (b) the nanopowder consists of liquid spherical nanoparticles; (c) conductive heat within the particles is smaller than the convective heat transfer at the surface particle, due to low Biot number (less than 0.1), and particle temperature could be considered uniform; (d) the radiative exchange from the plasma to particles, and between the particles themselves, are negligible because of low particle loading rates; (e) the effect of particle interaction amongst each other, and particles injection on the plasma jet characteristics, are not considered here, which is plausible in weak loading particles rate; and (f) the carrier gas velocity and the injection velocity of particles have the same value at the inlet plasma jet.

The evolution of particles position, velocity, and temperature in the plasma jet is simulated, using Lagrangian equations [47]. Therefore, the velocity of the particle is given by:

(17) $$m_{p}\frac{du_{p}}{\mathrm{dt}}=F_{D}+F_{g}+F_{X}$$

The particle’s position is given as:

(18) $$\frac{dx_{p}}{\mathrm{dt}}=u_{p}$$

where m_p_, **x_p_**, t are particle mass, location, and time, respectively, and $u_{p},\;F_{g},\;F_{X}$, and $F_{D}\;$are particles velocity vector, gravity force, external force, and drag force vectors, respectively.

The drag force is defined as:

(19) $$F_{D}=\frac{1}{\tau_{p}}m_{p}\left(u-u_{p}\right)$$

where $\tau_{p}$ is the response time of particle velocity, and u denotes the plasma flow velocity vector [48]:

(20) τp=4ρpdp23μCDRer 

where $\rho_{p}\;$and $d_{p}$ are the particle density and particle diameter, respectively. The Reynolds’ number Rer is given by:

(21) Rer=ρ‖u−up‖dpμ

$C_{D}\;$is an empirical function depending on the morphology of the particle, expressed as [49]:

(22) CD=24Rerf (Rer)

The function Rer is given by:

(23) f(x)=1,Rer≤0.21+0.1Rer0.99,0.2<Rer≤2.01+0.11Rer0.81,2<Rer≤21.01+0.189Rer0.63,21.0<Rer≤5000.44,Rer>500

The collisions between particles themselves, as well as with the injector wall, disperse the particle jet at the injector exit. Therefore, the fluid velocity applied for the drag force while turbulent dispersion is activated is written as:

(24) $$u=U+u^{\prime }$$

Here, U denotes the average velocity, and $u^{\prime }\;$represents the turbulent fluctuation:

(25) $$u^{\prime }=\xi \sqrt{\frac{2K}{3}}$$

ξ is a normally distributed random number with zero mean and unit standard deviation, and K is the turbulent kinetic energy.

The force of gravity force is represented by:

(26) $$F_{g}=m_{p}g\frac{\left(\rho_{p}-\rho \right)}{\rho_{p}}$$

g is the gravity vector, while $\rho_{p}$ represents the particles density.

The external forces $F_{X}$ include the thermophoresis force due to the gravity force, and the high thermal gradient in the background fluid, considered low in comparison with the drag force [50]. The thermophoretic force is given by:

(27) $$F_{t}=\frac{-6\pi d_{p}\mu^{2}C_{s}\Lambda \nabla T}{\rho (2\Lambda +1)T}$$

(28) $$\Lambda =\frac{\kappa }{\kappa_{p}}\;$$

where T is the fluid temperature, and ρ is the fluid density. κ and $\kappa_{p}\;$are the thermal conductivity of the fluid and the particle thermal conductivity, respectively. $C_{s}\;$is a constant value, equal to 1.17.

Thermophoretic force makes particles move from higher to lower temperature regions, and stabilize in the corner of the computational domain.

The particle temperature can be determined from the heat balance Equation [51]:

(29) $$m_{p}C_{p_{p}}\frac{{\mathrm{dT}}_{p}}{\mathrm{dt}}=Q_{\mathrm{conv},p}+H_{f}\frac{{\mathrm{dm}}_{p}}{\mathrm{dt}}$$

where $T_{p}\;$is the particle temperature, $C_{p_{p}}\;$is the specific heat of particles, and $H_{f}\;$is the latent heat assigned to the phase change.

The convective heat transfer at the surface of the particle is expressed as:

(30) $$Q_{\mathrm{conv},p}={\mathrm{hA}}_{p}\left(T-T_{p}\right)$$

h is the heat transfer coefficient between the plasma gas and the particle, $A_{p}$ is the particle surface area, and T is the temperature of surrounding fluid at the particle’s position.

The heat transfer coefficient could be written using the Nusselt number:

(31) $$h=\frac{\kappa \;\mathrm{Nu}}{L_{c}}$$

where $L_{c}$ is the characteristic length, typically the ratio of particle volume to particle surface area, and the Nusselt number is given by:

(32) Nu=(2+0.6Rep1/2Prf1/3)

where ${\mathrm{Pr}}_{f}\;$is the Prandtl number defined as:

(33) $${\mathrm{Pr}}_{f}=\frac{C_{p}\mu }{\kappa }$$

The thermophysical properties of silicon powders, reported in [52,53], are used in the implementation of the model, and are illustrated in Table 2.

**Figure 5:**
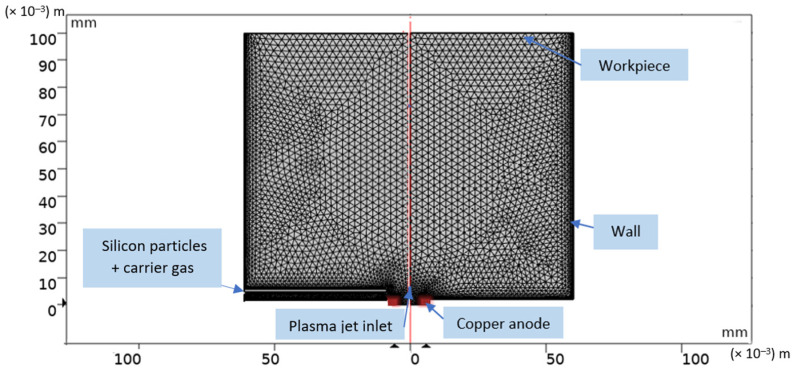
Computational domain of reactor chamber.

The turbulent thermal plasma jet transporting silicon nanopowder is simulated, using a computational fluid dynamic module and particle tracing module in three dimensions, via COMSOL Multiphysics 5.4 [54]. The Galerkin finite element method is employed for discretizing the governing equations. The size of the jet domain used for the calculation is 60 mm and 100 mm in the radial and axial directions, respectively. A non-uniform mesh size is used to have more accuracy in our results. The total grid number in the computational domain is found at about 3782. Figure 5 illustrates the computational domain of reactor chamber used for the simulation of the particle-plasma jet. The COMSOL simulations were performed on an Intel^®^ Core (TM) i7-HP computer, with 1.99 GHz and 16 GB of installed memory (RAM), running a Windows 10 with 64 bit operating system. Simultaneous solutions of time-dependent coupled equations took 15 h 10 min.

## 4. Numerical Results

### 4.1. Validation

**Figure 6:**
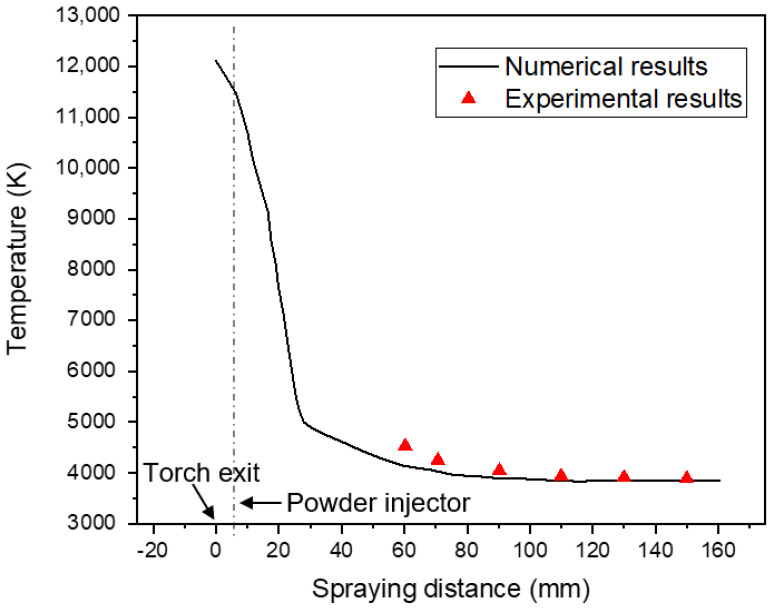
Calculated and experimental measurement of plasma jet temperature profiles at the centerline axis as a function of spraying distance.

In Figure 6, the calculated plasma jet temperature along the centerline axis are compared with experimental measurements taken from literature [55] using the same operating conditions (450 A, Ar/H_2_ (40/10 lpm)). The calculated temperatures are in good agreement with associated experimental measurements, which allows the validation of our model.

Following the plasma jet’s exit from the torch, the temperature profile shows a downward trend. The first rapid decline occurs due to the expanding process of plasma jet, no longer constrained by the torch nozzle. The second significant decline is associated with the shilling of the cold entrainment gas and cold powder carrier gas into the plasma jet central region.

### 4.2. Plasma Jet Distribution

**Figure 7:**
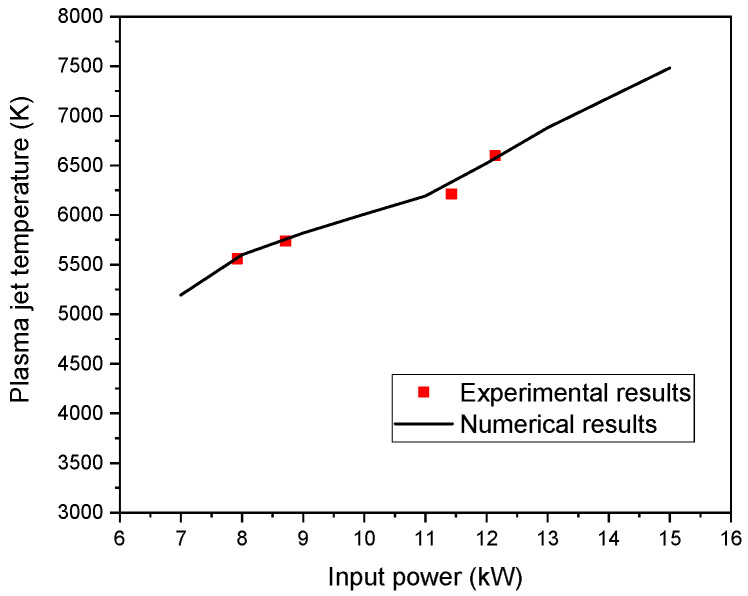
Variation of calculated and experimental input plasma jet temperature with input power.

Numerical results of a turbulent plasma argon jet emerging in an argon atmosphere with particle injection are presented here. As a precursor material, silicon particles of 20 µm average diameter are freed simultaneously with argon gas in the plasma flame. Carrier gas and plasma mass flow ratio are assumed as 0.06 and 0.2 for an injection gas speed of 10 or 30 m/s, respectively. At first, the variation of calculated plasma jet temperature as a function of input power are presented in Figure 7 and are compared with other experimental works [56] using the same operating conditions, in order to be confident that the model is robust against small variations of input plasma parameters.

**Figure 8:**
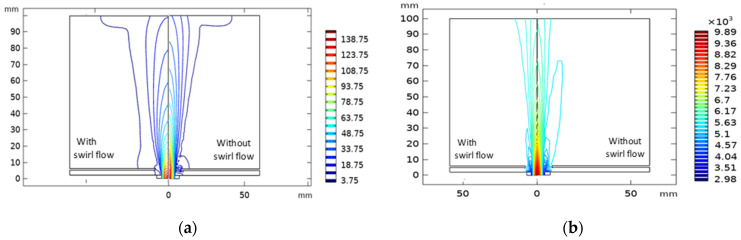
Swirl flow impact on: (**a**) the field velocity distribution, (**b**) the computed isotherms of the turbulent plasma jet.

The swirl flow effect on velocity and temperature plasma field is represented in Figure 8. When admitting a swirl flow, the vortex and the bubbles close to the carrier gas inlet mostly disappear, and the plasma gas flow becomes almost uniform (see Figure 8a). Simultaneously, in temperature distribution, the vortex size decreases in the presence of swirl velocity, while the rate of radical convection heat transfer towards the reactor edge increases, due to the high radial velocity (see Figure 8b). Furthermore, the velocity and temperature drop along the jet axis towards the substrate, because of the corresponding swirling velocity at the jet inlet [57].

**Figure 9:**
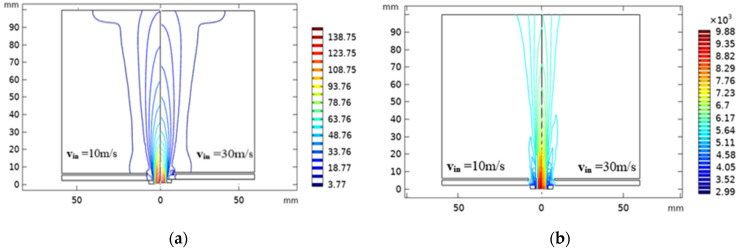
(**a**) Velocity field distribution and (**b**) computed isotherms of the turbulent plasma jet with swirl flow for different injection particles velocities (v_in_ = 10 m/s and v_in_ = 30 m/s).

Plasma field velocity and temperature under different injection velocities are illustrated (v_in_ = 10 m/s and v_in_ = 30 m/s; see Figure 9). The increase of particle injection speed reduces the velocity distribution, as well as the temperature field around the plasma jet axis, especially in the upstream region of the inlet plasma flame where the phase change of particles occurs actively. This result is due to an elevated energy transfer between the fluid and the particles in the vicinity of the central jet axis with the increase in the particles’ feed rate [58].

### 4.3. Particles Distribution

**Figure 10:**
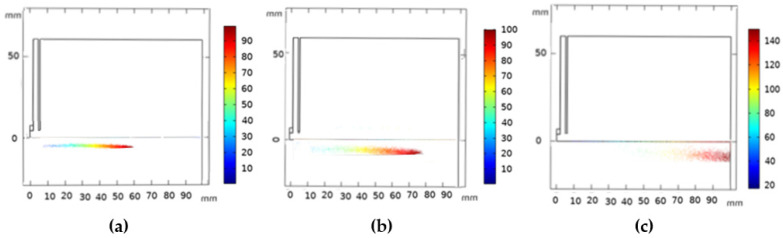
Predicted particles distributions at different instants (t) under the plasma jet. (t = 1.5 ms (**a**), t = 2 ms (**b**), t = 3 ms (**c**)).

We investigated the particle’s motion and their thermal history characteristic, such as path, velocity, acceleration, surface temperature, and heat flux. These parameters are important due to their high impact on the nanoparticle’s quality [59]. The spatial distribution of silicon particles (N_p_ = 10,000, d_p_ = 20 µm, and v_in_ = 20 m/s) injected in the plasma jet at different instants is studied (see Figure 10). Particles are accelerated by the carrier gas, and combine with the plasma stream to form an injection channel, where they deeply penetrate, resulting in a higher gas temperature during their motion.

**Figure 11:**
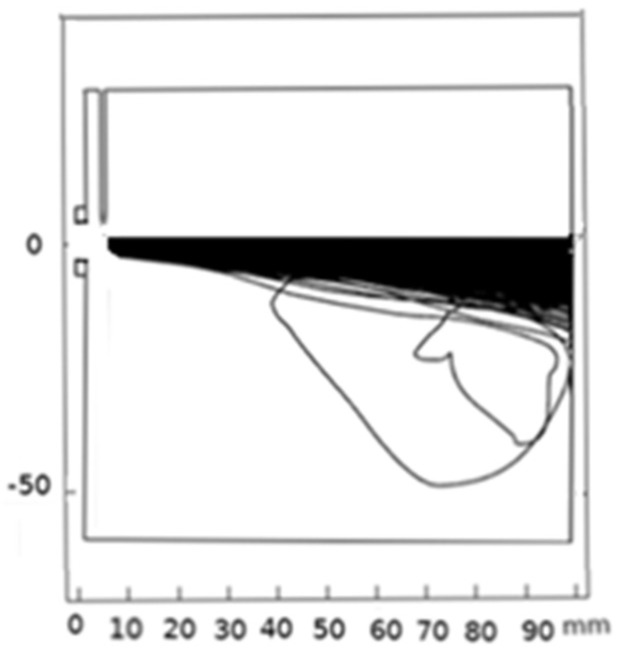
Silicon particles trajectory along the plasma jet (N_p_ = 10,000, d_p_ = 20 µm, and v_in_ = 20 m/s).

The main trajectory of particles inside the plasma jet is illustrated in Figure 11. The injected particle’s trajectory is found moving from the inlet port into the substrate. However, a portion of the injected particles avoids the substrate by flowing backward. This is due to the strong particle’s interaction with the fluid flow. Herein, fluid streamlines running near the center axis bend sharply at the front of the substrate [60].

### 4.4. Influence of Parametric Study on Characteristic of Particles Distribution

The quality of nanoparticles formed is highly related to the injection carrier gas velocity, particles (diameter, number), and jet characteristics.

#### 4.4.1. Injected Particle Velocity

**Figure 12:**
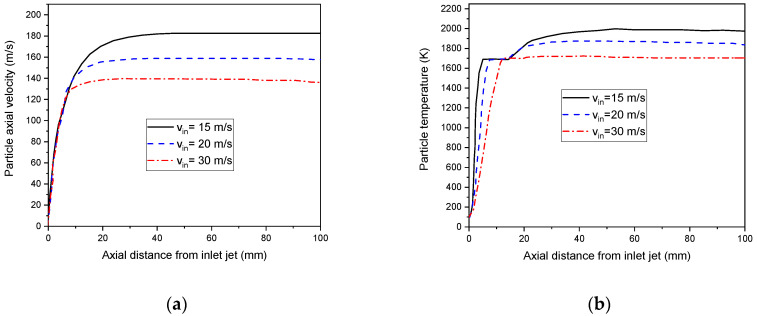
Axial velocities (**a**), surface temperature (**b**) of a 20 µm particles under different injection carrier gas velocities (v_in_ = 15 m/s, 20 m/s, and 30 m/s).

The impact of the injected carrier gas on the particle velocity and temperature distribution travelling along with the plasma jet at speeds of 15, 20, and 30 m/s are studied (see Figure 12). We demonstrate that a higher axial velocity of particles ($u_{p}$) are achieved under the flow of weak-injection carrier gas velocities. The particles are located mainly at the central portion of the flame.

This result could be assigned to particle acceleration with the axial direction if the small effect of the external forces $F_{X}\;$is neglected [61]:

(34) dupdt=18μρdp2(u−up) f(Rer)

Since f(Rer) takes a positive value, the acceleration sign is proportional to the plasma jet speed and the particle velocity difference. At higher-injection carrier gas velocity, the acceleration of particles decreases. Consequently, they exceed the central area to reach the slower external part of the plasma jet. However, no acceleration could be located when the particle and plasma jet velocity are identical.

Figure 12b displayed the effect of injection carrier gas velocity on the temperature of s particles. The results demonstrate that an increase in injected carrier gas velocity, from 15 m/s to 30 m/s, decreases the highest particle temperature [62].

#### 4.4.2. Particle Size

**Figure 13:**
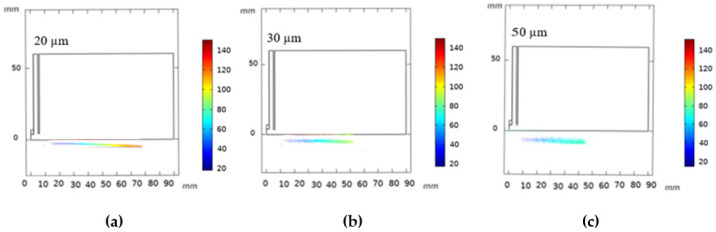
Trajectories of the silicon particles at the same instant with different particle diameters in the plasma jet (d_p_ = 20 µm (**a**), d_p_ = 30 µm (**b**), and d_p_ = 50 µm (**c**)).

The path of silicon particles at the same instant (N_p_ = 10,000, v_in_ = 20 m/s) with variable diameters (20 µm, 30 µm, 50 µm) is shown in Figure 13. Smaller particles easily accelerate in the flame and gain higher axial velocity. This could be explained with Equation (34), where both density and the squared particle diameter are inversely proportional to the acceleration. However, large size particles owning greater inertia tend to fly away from the flame center, where the gas velocities are much lower, and attain lower velocities [63].

**Figure 14:**
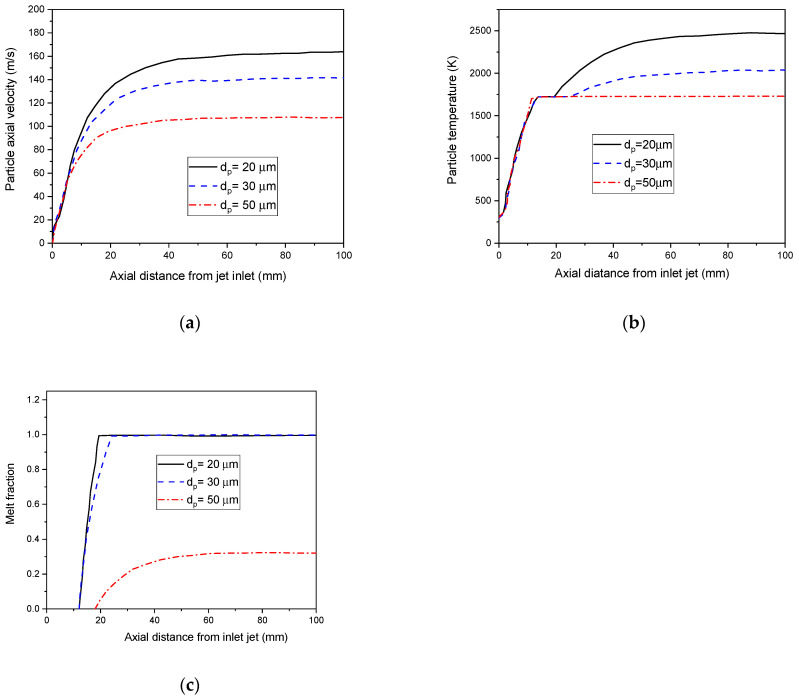
Axial velocities (**a**), surface particle temperature (**b**), and melt fraction (**c**), with different particles diameters in the plasma jet (10 µm, 20 µm, 30 µm).

Figure 14a represents the particle’s axial velocities with different particle diameters. Indeed, all particles accelerate at the beginning to gradually reach a uniform velocity. The particles with a 20 µm diameter own a higher temperature surface (see Figure 14b). However, particles of 50µm or greater remain below their melting point (1685 K) in a plasma jet (see Figure 14c). This is due to the significant amount of energy required to melt the particles. Indeed, a good coating quality requires a melting extension of inflight particles [64].

#### 4.4.3. Particles Number

**Figure 15:**
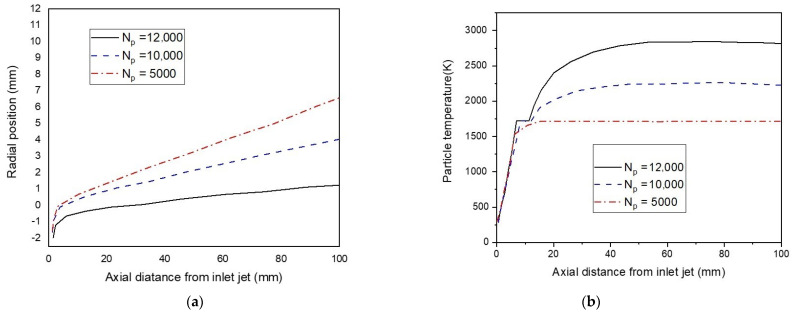
The radial trajectory of particles (**a**) and the particle’s temperature (**b**), under a different number of particles (N_p_ = 5000, 10,000, and 12,000).

Figure 15 represents the particle’s path and temperature with several numbers of particles (N_p_ = 3000, 5000, and 10,000). For a higher number of particles, total momentum and energy exchanges increase, and the injected particles assume deeper penetration during their motion in the gas channel (see Figure 15a). This then results in a higher particle temperature. (Figure 15b).

#### 4.4.4. Plasma Flow Rate

**Figure 16:**
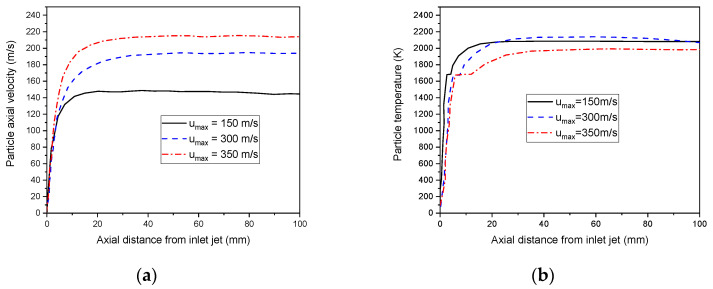
The axial particle velocity (**a**), and the particle temperature (**b**) distributions, under different initial velocities plasma flow rate (u_max_ = 150, 300, and 350 m/s).

The variation of the plasma flow may highly affect the momentum and temperature of the particles, as illustrated in Figure 16. The effect of the change in inlet plasma gas velocity on the particle’s velocity is shown in Figure 16a. For high gas velocities, particles gaining high speed increases are attracted to the central axis, owing to their lower pressures. The radial particle velocities then decrease. Figure 16b describes the influence of initial jet velocity on the particle’s temperature. Indeed, particles reach the melting point (1685 K) with different residence times in the plasma jet. A high-velocity jet confines particles in the core flame and minimize their residence. Herein, particles own an elevated heat transfer coefficient, which consequently improves the thermal efficiency between the flame and the particle surface.

**Figure 17:**
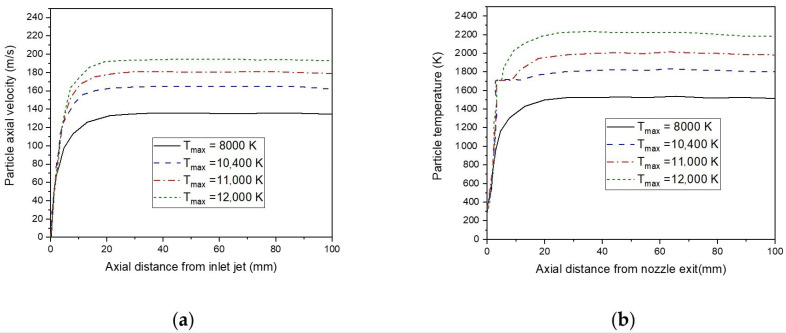
The axial particle velocity (**a**), and the particle temperature (**b**) distributions, under different initial temperatures plasma jet (T_max_ = 8000, 10,400, 11,000, and 12,000 K).

Here, various initial plasma temperatures are predicted by the model, and their effects are shown in Figure 17. The particle temperature increases at high temperature of plasma gas, even when they have a short residence time with high velocities. Indeed, a plasma temperature of 8000 K cannot melt the particles of silicon. Raising the temperature of plasma flow is beneficial for particle temperature because it increases the heat transfer coefficient, in addition to increasing the relative particle velocity.

#### 4.4.5. Particle Turbulent Dispersion

**Figure 18:**
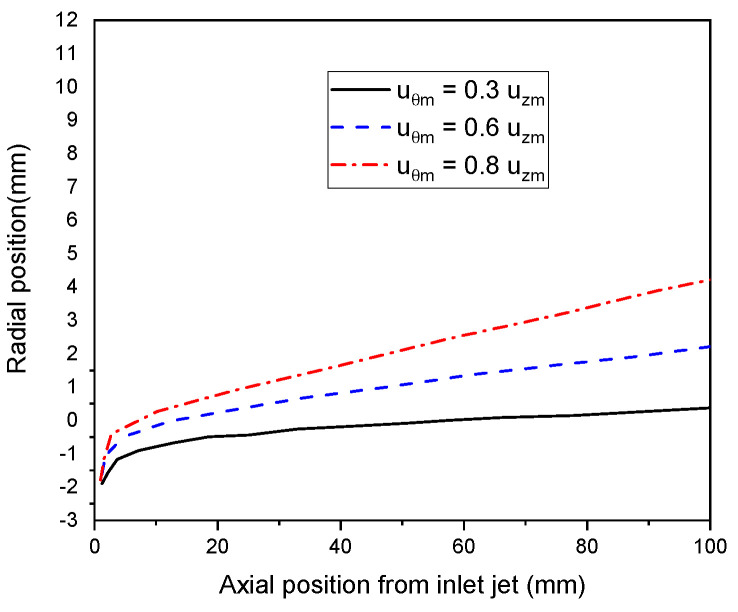
Trajectory of particles at different intensity of swirl flow of plasma jet (u_θm_ = 0.3 u_zm_, u_θm_ = 0.6 u_zm,_ and u_θm_ = 0.8 u_zm_).

Here, the trajectories of the sprayed particles at different intensities of swirl flow (u_θm_ = 0.3 u_zm_, u_θm_ = 0.6 u_zm,_ and u_θm_ = 0.8 u_zm_) are illustrated in Figure 18. Due to random turbulent fluctuation, particles with the same conditions of diameter, injection velocity, and injection time have various paths and temperature histories. Turbulent dispersion widens the particle’s trajectories and heating histories and may hide the effect caused by the jet flow field. Indeed, due to the inlet swirling flow, the temperature and velocity drop along the axial jet axis towards the substrate. Momentum and transferred energy from plasma to particles decrease, defecting the particle’s trajectory away from the plasma jet axis at higher swirl velocity.

## 5. Conclusions

The effect of an injection of Si particles on the non-transferred turbulent argon plasma jet is investigated, using a three-dimensional numerical model. Plasma thermo-fluid dynamics, precursor-paths, particle growth process, and thermal history are described using a simulation model. The effect of different precursor flow rates, diameters, and the numbers of particles are examined, to determine the optimal growth conditions. The swirling flow reduces the localized vortex, and decreases transferred energy and momentum in the plasma jet. Consequently, particles deviate away from the plasma jet’s centerline. Temperature and velocity flow fields are strongly deformed at elevated carrier gas flow rates. An injection carrier gas velocity of 10 m/s is insufficient for the particles to be completely melted, while with an injected carrier gas velocity greater than 20 m/s, particles overshoot the central zone, and reach the outer slower region of the jet. The particle’s temperature and axial velocity vary drastically along the jet’s axis with the different sizes and numbers of the particles. At smaller diameters, particles attain higher terminal velocities, and accelerate through a longer flight distance. However, particles larger than 50 µm diameter remain below their melting point when sprayed. This is because, in this case, a larger amount of energy is needed to melt the particle. Furthermore, the initial plasma field distribution at the upstream flame influences the magnitude of the particle’s temperature and velocity distribution. A high-velocity plasma jet provides a short residence time, allowing the particle to be pulled into the hot core of the flame, and increases the heat transfer efficiency from the flame to the particle surface. Raising the plasma gas temperature increases the particle temperature strongly, despite the reduced residence time, due to high velocities. A plasma temperature of 8000 K is insufficient to melt the silicon particles.

Therefore, thermal plasma plays a major role in controlling high-enthalpy flow, rapid quenching, and the ability to synthesize high-quality nanoparticles. Adjusting the optimal parameters, such as injection velocity of carrier gas about 20 m/s, particles with diameter 20–40 µm, thermal plasma jet issuing from non-transferred plasma torch with a relative velocity of 150 m/s, and relative temperature greater than 8000 K, as well as adding swirling flow to control the plasma jet fields deflection, suppresses locally strong deformation.

## Figures and Tables

**Table 1:** Operational parameters used for silicon nanoparticles synthesis.

Operating Mode	Non-Transferred Plasma Arc
Plasma current	100–200 A
Plasma voltage	80–200 V
Working gas	Argon
Plasma flow rate	20 lpm
Plasma power range	8–30 kW
Outlet electrode diameter	8 mm
Powder carrier gas flow rate	1.2–3 lpm
Powder feed rate (silicon)	9 g/min
Quenching medium	Argon
Working gas pressure	1 atm

**Table 2:** Thermophysical characteristics of silicon particles.

Parameters	Value
Molecular weight	28.0855 g/mol
Melting point T_melt_	1685 K
Boiling point T_boil_	3504.616 K
Specific heat $C_{p_{p}}$	0.71 J/g K
Mass density $\rho_{p}$	2329 kg/m^3^
Thermal conductivity $\kappa_{p}$	148 W/m/k
Enthalpy of fusion	1787.75 kJ/kg
Enthalpy of vaporization	13,690.3 kJ/kg

## Data Availability

Data are contained within the article.
